# Mechanical Shunt Resonators-Based Piezoelectric Metamaterial for Elastic Wave Attenuation

**DOI:** 10.3390/ma15030891

**Published:** 2022-01-24

**Authors:** Jiawen Xu, Hang Lu, Weiyang Qin, Ping Wang, Jie Bian

**Affiliations:** 1 Jiangsu Key Lab of Remote Measurement and Control, School of Instrument Science and Engineering, Southeast University, Nanjing 210096, China; 220203539@seu.edu.cn; 2 Department of Engineering Mechanics, Northwestern Polytechnical University, Xi’an 710072, China; qinweiyang@nwpu.edu.cn; 3 AECC Hunan Aviation Powerplant Research Institute, Zhuzhou 412002, China; Bianjie_hrbeu@163.com; 4 AECC Key Laboratory of Aero-Engine Vibration Technology, Zhuzhou 412002, China

**Keywords:** piezoelectric metamaterial, piezoelectric transducer, mechanical shunt resonator, elastic wave attenuation

## Abstract

The conventional piezoelectric metamaterials with operational-amplifier-based shunt circuits have limited application due to the voltage restriction of the amplifiers. In this research, we report a novel piezoelectric metamaterial beam that takes advantage of mechanical shunt resonators. The proposed metamaterial beam consisted of a piezoelectric beam and remote mechanical piezoelectric resonators coupled with electrical wires. The local resonance of the remote mechanical shunt resonators modified the mechanical properties of the beam, yielding an elastic wave attenuation capability. A finite-length piezoelectric metamaterial beam and mechanical shunt resonators were considered for conceptual illustration. Significant elastic wave attenuation can be realized in the vicinity of the resonant frequency of the shunt resonators. The proposed system has the potential in the application of wave attenuation under large-amplitude excitations.

## 1. Introduction

Phononic crystals and metamaterials have attractive potential in elastic wave manipulation and attenuation [1,2,3,4,5,6,7,8,9,10,11,12,13,14,15,16,17,18,19,20,21,22,23,24,25,26,27,28,29]. They show promising advantages in the applications of negative refraction [2,3], acoustic cloaking [4,5], wave focusing [6,7,8,9,10], wave attenuation [11,12,13,14,15,16,17], and vibration-mode tailoring [18]. The features of phononic crystals and metamaterials stem from Bragg scattering and local resonance, respectively [19,20,21,22,23]. In particular, the Bragg scattering phenomenon occurs, when the length of the elastic wave is at the same level as the dimension of the unit-cell [24,25,26]. Since the wavelength is roughly inversely proportional to the frequency of the elastic wave. It is necessary to adopt large-size unit-cells for the application in the low-frequency regime. On the other hand, metamaterials take advantage of the local resonance behavior of integrated local resonators, i.e., the system dynamics does not rely on the unit-cell dimension, and possess the extraordinary capability of low-frequency elastic waves manipulation with small-size unit-cells [27,28,29].

Metamaterials-based elastic wave attenuation has attracted extensive attention. For example, metamaterials were utilized for the vibration control in structural bars and beams [12,25,30,31]. In addition, it was demonstrated that the width of the bandgap of metamaterials, i.e., the frequency range of wave attenuation, is expandable by structural parametric optimization [12]. It is worth mentioning that the frequency range of wave attenuation is expandable by adopting rainbow resonators [15,16]. In the past, the metamaterials were assembled by local mechanical resonators integrated into primary structures [12,25,30,31]. The systems feature strong wave attenuation within a fixed frequency range [12,30,32]. Typically, additive local resonators are attached to primary structures. Such a design requires extra construction volumes for resonators [12], and the compactness of the systems cannot be maintained. On the other hand, researchers embedded subtractive micro-resonators into the main structure to build mechanical metamaterials. However, this type of metamaterial suffers from a significant stiffness reduction [32]. Ideally, a metamaterial is capable of attenuating elastic waves in a low-frequency range without changing the primary structure or requiring extra construction volumes.

To introduce online tunability, piezoelectric transducers were adopted due to their two-way electro-mechanical coupling [13,33]. Piezoelectric phononic crystals take advantage of Bragg scatting. Piezoelectric metamaterial beams and electromechanical beams adopt local resonance behaviors. In a typical piezoelectric metamaterial system, inductive shunt circuits are connected to piezoelectric transducers that can directly produce a local resonance. For instance, periodic piezoelectric transducers with individually connected inductance shunt circuits are integrated into a rod for wave attenuation [26,34]. In addition, it was demonstrated experimentally that a piezoelectric metamaterial beam is capable to attenuate elastic waves by −50.7 dB [33]. Adjusting the shunt circuit can effectively modify the bandgap behavior of piezoelectric metamaterials without modifying the mechanical structures. Piezoelectric metamaterials have advantages over mechanical ones due to their simple configuration and adaptivity. In other words, it is possible to realize strong wave attenuation without changing the primary structure [13,26,34]. Recently, Chen et al. demonstrated that tunable, topologically protected interface modes in soft membrane-type metamaterials can be established using piezoelectric phononic crystals [35]. In addition, Edson et al. investigated the dispersion relation of one to three piezoelectric phononic structures with Langasite cylindrical inclusions [36]. It was claimed that piezoelectricity enhances the bandgap widths and yields a bandgap in lower frequencies. Qian et al. investigated the first-order bandgap properties in a nanoplate by analyzing the influences of the residual surface stress and the material intrinsic length [37].

However, creating a low-frequency local resonance in piezoelectric metamaterials requires inductance shunt circuits with a value up to tens of Henry. Such a large inductor is difficult and sometimes impossible to be built passively. Hence, active inductance shunt circuits are introduced using operational amplifiers (op-amps) [26,33]. However, the working range of shunt circuits is limited by the voltage limitation of op-amps and voltage supplies. On the other hand, we may need to suspend large-amplitude waves in real applications. In such a case, large-amplitude excitations may yield a large-amplitude voltage in a shunt circuit. The voltage may break op-amps, as they can hold voltages no more than tens of volts [38]. For example, the op-amp TL082 adopted in reference [33] has a voltage limitation of ±15 V. Besides, the high-voltage op-amp designed for the application of piezoelectric transducer, OPA445, has a voltage limitation of ±45 V. Hence, the applications of piezoelectric metamaterials are limited in real applications. It is worth noticing that piezoelectric ceramics plates are capable to hold voltages up to thousands of volts. In other words, the limitation of conventional piezoelectric metamaterials would be overcome, if we re-design shunt elements. 

In this research, using the theory of local-resonance-based metamaterials, a mechanical-resonator-based piezoelectric metamaterial was proposed to overcome the voltage limitation in conventional systems. Here, op-amps-based shunt circuits in conventional piezoelectric metamaterials were replaced by mechanical shunt structures to avoid the issues of voltage limitations. We hypothesized that the local resonance of remote mechanical resonators can effectively modify the equivalent material property of a piezoelectric beam, yielding wave attenuation. Theoretical illustration and finite element method (FEM) simulations were carried out to demonstrate the wave attenuation features of the proposed system. We also analyzed the influences of the proof masses of the mechanical resonators on the wave attenuations. The proposed concept has many applications, including wave attenuation and vibration suspending in machines, vibration isolation platforms, and precision measurement.

## 2. Conceptual Illustration

**Figure 1:**
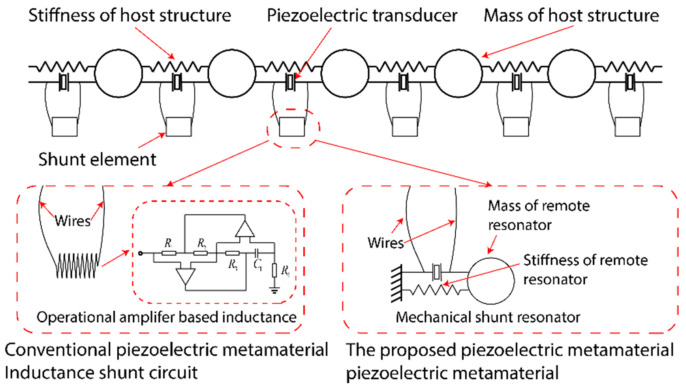
Conceptual illustration. The analogy shunt circuits are replaced with remote mechanical shunt resonators.

The research focuses on subwavelength elastic wave attenuation using a local-resonance-based metamaterial. The conceptual illustration of the one-dimensional piezoelectric metamaterial is shown in Figure 1. The metamaterial consists of the main structure and multiple remote shunt elements. Piezoelectric transducers are attached to the main structure and the remote ones. In the conventional piezoelectrical metamaterial, op-amp-based shunt circuits are adopted as the shunt elements. In the proposed system, we replaced the op-amp-based circuits with mechanical shunt resonators to overcome the voltage limitation. Specifically, the piezoelectric transducers on the main structure are connected individually to the piezoelectric transducers attached to the remote resonators. Hence, the host structure and the mechanical shunt resonators are coupled by the two-way electromechanical coupling of the piezoelectric transducers. 

The underlying physics is that the elastic waves propagating through the main structure may induce a resonant motion of the mechanical shunt resonator due to the electromechanical coupling. The resonant motion would then affect the wave propagation in the main structure in return. Consequently, the wave attenuation effect can be achieved. Notably, the proposed design has advantages over conventional mechanical or piezoelectric metamaterials in several aspects. Firstly, the proposed system does not require large extra construction volumes for local resonators. In addition, it would not reduce the overall stiffness of the structure. Secondly, the proposed design overcomes the limitation of the voltage in op-amp-based inductive shunt circuits, i.e., it has the potential in attenuating large-amplitude elastic waves. It is worth mentioning that, all the mechanical shunt resonators do not need to have the same size. Local resonators with graded frequencies may yield an enhanced wave attenuating capability [39].

**Figure 2:**
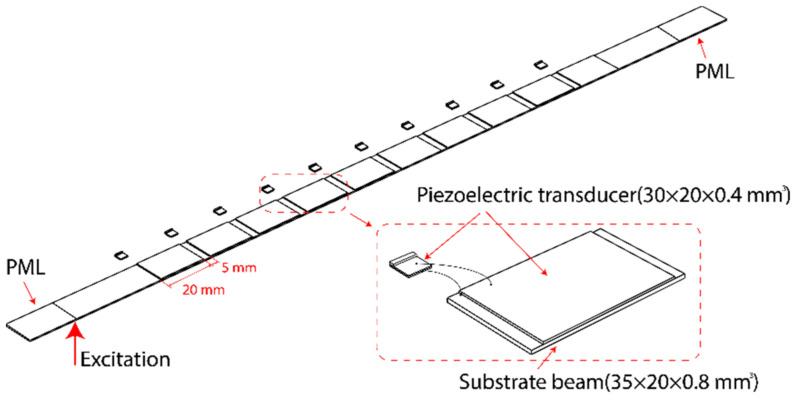
Metamaterial beam with mechanical shunt resonators.

We applied the concept to design a piezoelectric metamaterial beam. Without loss of generality, a slender piezoelectric beam was adopted as the main structure, as shown in Figure 2. Piezoelectric transducers were attached to the beam periodically. Mechanical shunt resonators made of piezoelectric cantilevers were placed in the vicinity of the main beam. The piezoelectric transducers on the main beam were connected individually to the one on the mechanical shunt resonators. In addition, two perfectly matched layers (PMLs) were integrated into the system at the two free ends of the main beam. In the following analysis, we focused on a transverse wave propagating in the longitude direction of the main beam. Here, we considered an elastic wave propagating from the left end to the right end. 

To investigate the features of the proposed system, the unit-cell level system dynamics is illustrated. The equivalent Young’s modulus of the piezoelectric transducer on the main beam was given as [33,40,41]:

(1) $$E_{p}^{SU}=E_{p}^{D}\left(1-\frac{k_{31}^{2}}{1+i\omega C_{p}^{\varepsilon }Z^{SU}(\omega )}\right),$$

where $E_{p}^{SU}$ is the equivalent Young’s modulus of the piezoelectric transducer, $E_{p}^{D}$ is the Young’s modulus of the piezoelectric transducer under a constant electrical displacement, ω is the frequency of excitation, $k_{31}$ is the electromechanical coupling coefficient of the piezoelectric transducer, $C_{p}^{\varepsilon }$ is the capacitance of the piezoelectric transducer, $Z^{SU}\left(\omega \right)$ is the equivalent electrical impedance of the mechanical shunt resonator. Here, the mechanical dynamics of the mechanical shunt resonator were modeled as an electrical impedance. Hence, it is necessary to obtain the equivalent impedance of the mechanical shunt structure. The equivalent impedance can be solved from the electromechanical coupling model of the mechanical shunt resonator. The lumped model of the shunt structure was given as [42]:

(2) $$m\frac{d^{2}x}{dt^{2}}+c\dot{x}+kx+k_{12}Q=0,$$

(3) $$R\dot{Q}+\frac{1}{C_{p2}}Q+k_{12}x=V,$$

where *m, k* and *c* are the lumped mass, stiffness, and damping of the shunt resonator, respectively, $k_{12}$ is the electromechanical coupling coefficient of the resonator, Q is the charge on the piezoelectric transducer, $C_{p2}$ is the capacitance of the piezoelectric transducer, *V* is the voltage of the piezoelectric transducer, and *R* is the resistance in the wires. Notably, $k_{12}$ is the system-level electromechanical coupling coefficient of the shunt resonator rather than the coefficient of the piezoelectric transducer. The lumped parameters of the shunt resonator can be obtained using the assumed mode method [40]. Here, the shunt resonators were assumed to work in the first bending mode. It is worth noticing that Euler–Bernoulli’s beam theory was used in the modeling. Advanced theories, such as Timoshenko beam theory, could be applied for molding the higher-frequency performances of the system. In addition, all the shunt resonators had clamped-free boundary conditions. Substituting Equation (2) into Equation (3), we obtained the equivalent electrical impedance of a shunt resonator as:

(4) $$Z=\frac{V}{\dot{Q}}=R+\frac{1}{i\omega C_{p2}}-\frac{k_{12}^{2}}{i\omega \left(-\omega^{2}m+i\omega c+k\right)}.$$

Equation (4) indicates that the mechanical dynamics of the shunt resonators are included in the electrical impedance. In addition, the influences of the dynamics of the shunt resonator are proportional to the electromechanical coupling coefficient $k_{12}^{2}$ Moreover, the equivalent electrical impedance includes a component of the capacitance of the piezoelectric transducer $1/i\omega C_{p2}$ Substituting Equation (4) into Equation (1), the equivalent Young’s modulus of the piezoelectric transducer on the main beam can be given as:

(5) $$E_{p}^{SU}=E_{p}^{D}\left(1-\frac{k_{31}^{2}}{1+i\omega C_{P}^{\varepsilon }R+\frac{C_{P}^{\varepsilon }}{C_{p2}}-\frac{C_{P}^{\varepsilon }}{m}\frac{k_{12}^{2}}{-\omega^{2}+i\omega \;c/m+\omega_{0}^{2}}}\right),$$

where $\omega_{0}$ is the resonant frequency of the mechanical shunt resonator. The local resonance was calculated using the assumed mode method [40]:

(6) $$\omega_{0}^{}=\sqrt{k/m},$$

and

(7) $$m=2\rho_{p}A_{p}\int_{0}^{l_{p}}\varphi^{2}\left(x\right)dx+m_{pm}\varphi^{2}\left(l_{p}\right),$$

(8) $$k=2\int_{0}^{l_{p}}EI_{p}{\left(\varphi^{\prime }\right)}^{2}dx,$$

where $\rho_{p}$ is the density, $A_{p}$ is the mass per length, $l_{p}$ is the length, $m_{pm}$ is the proof mass, $\varphi \left(x\right)$ is the assumed mode shape, *E* is the Young’s modulus, and $I_{p}$ is the moment of inertial of the mechanical resonator. Equation (4) indicates that the equivalent Young’s modulus of the piezoelectric transducer on the main beam is a function of the resonance of the mechanical shunt resonator. In other words, coupling the shunt resonator via piezoelectric transducers can effectively introduce the local resonance effect to the main beam. In such a case, the dispersion relation of the main beam would vary significantly in the vicinity of the resonant frequency of the shunt resonator [33,40,41]. Hence, the wave attenuation effect is introduced. In addition, the equivalent Young’s modulus of the piezoelectric transducer on the main beam contains components of $C_{p}^{\varepsilon }/C_{p2}$ and $C_{p}^{\varepsilon }/m$. 

## 3. Parameter Selection of the Shunt Resonator

**Figure 3:**
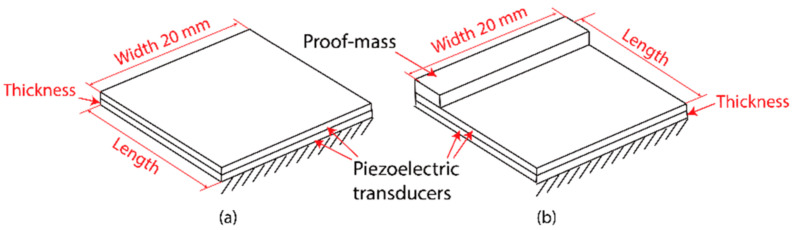
Configurations of the mechanical shunt resonators: (**a**) without a proof mass; (**b**) with a proof mass.

In this section, we proceed to the parameter selection of the mechanical shunt resonators. Here, we considered cases where the shunt resonators without and with a proof mass to investigate the features of the system, as shown in Figure 3. 

**Figure 4:**
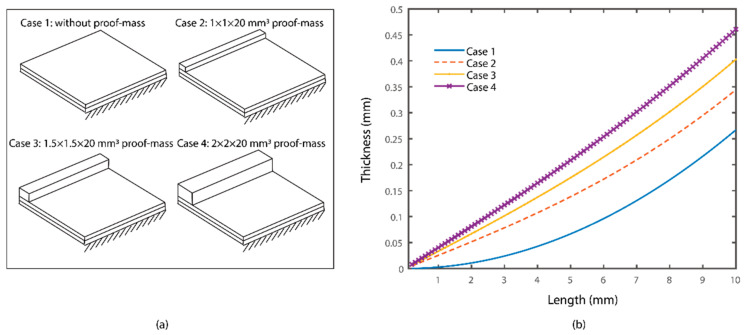
(**a**) Configurations of the mechanical resonators in four cases; (**b**) parameters combinations of the shunt resonators.

The configurations of the mechanical shunt resonators are shown in Figure 3. The resonators contained a bimorph piezoelectric cantilever with clamped-free boundary conditions. Several cases were considered, where the shunt resonators did or did not have proof masses to the main beam. To evaluate the system dynamics under the same circumstance, the parameters of the resonators with the same resonance frequency were selected. Without the loss of generality, 3 kHz was chosen as the working frequency of the resonators for the conceptual illustration. It is worth mentioning that another frequency point can be chosen as the working frequency can be modified by adjusting the parameters of the mechanical resonators. Here, four cases were considered as following: case 1, the resonators had no proof mass; case 2, the resonators had a copper proof mass with a dimension of 1 × 1 × 20 mm^3^; case 3, the resonators had a copper proof mass with a dimension of 1.5 × 1.5 × 20 mm^3^; and case 4, the resonators had a copper proof mass with a dimension of 2 × 2 × 20 mm^3^. The resonant frequencies of the resonators were tuned by adjusting their lengths and thicknesses. The parameters combinations for the resonators in the four cases are presented in Figure 4.

Figure 4 shows the four cases and the parameters combinations of the mechanical shunt resonators. It can be obtained that the thickness of one layer of the resonators increased with the increase of the length to create a local resonance at 3 kHz. Besides, an enlarged proof mass required a larger thickness of the resonators. Without loss of generality, the lengths of the shunt resonators were chosen as 6.25 mm, 7.5 mm, 8.75 mm, and 10 mm in the following analysis. The thicknesses of the resonators can be obtained from Figure 4b. Besides, the widths of the resonators was chosen as 20 mm. It is worth noticing that the resonant frequencies of the resonators were independent of the widths according to Euler beam theory. Moreover, the configuration of the primary structure was kept the same in this research.

## 4. Wave Attenuation Characteristics

**Figure 5:**
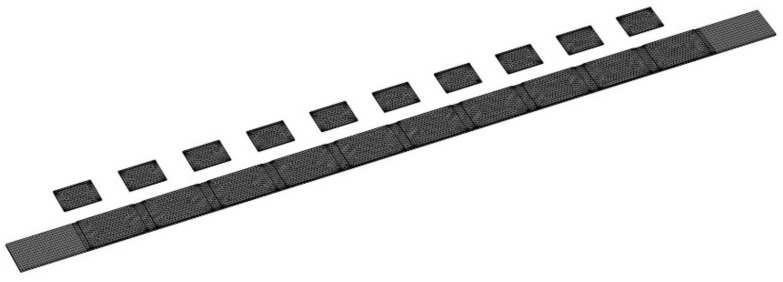
Mesh of the finite-element method (FEM) model.

In this section, we analyzed the wave attenuation features of the proposed system in the vicinity of the resonant frequency of 3 kHz. Here, the main beam had a dimension of 350 × 20 × 0.8 mm^3^. The main beam had a density, a Young’s modulus, and a Poisson’s ratio of 7850 kg/m^3^, 200 GPa, and 0.3, respectively. Piezoelectric transducers PZT-5H were chosen. The piezoelectric transducers attached to the main beam had a dimension of 30 × 20 × 0.4 mm^3^. Ten piezoelectric transducers were glued onto the main beam periodically with an interval of 5 mm. The density, the Young’s modulus, the piezoelectric coefficient, and the dielectric coefficient of the piezoelectric transducer were 7500 kg/m^3^, 127 GPa, −2.74 × 10^−10^ C/N, and 1433.6, respectively [17,25,31]. In the following analysis, excitation was applied on one end of the main beam, and the response displacement was obtained on the other end. The FEM is widely employed in the field of acoustic metamaterials and phononic crystals, as it can match well experimental studies in piezoelectric metamaterial investigations [22,30,33,34]. FEM software COMSOL 5.5 was adopted in the analysis. The mesh of the FEM model is presented in Figure 5.

**Figure 6:**
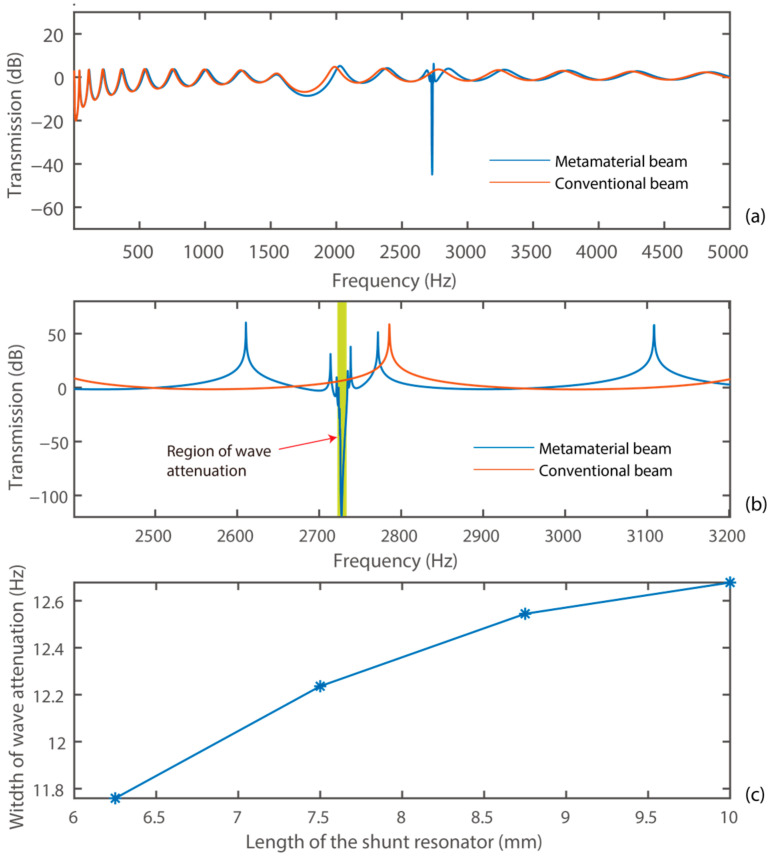
Characteristics of the system without a proof mass: (**a**) transmission diagram; (**b**) zoom−in transmission diagram in 2400–3200 Hz; (**c**) frequency width of wave attenuation.

In the simulation, the default element type was chosen. In addition, the number of elements was 64,468. The transmission diagram of the integrated system was calculated as [12,13,33]:

(9) $$Trans(\omega )=20\log_{10}\frac{A_{trans}(\omega )}{A_{in}(\omega )},$$

where $A_{in}(\omega )$ is the amplitude of the incident elastic wave, and $A_{trans}(\omega )$ is the amplitude of the transmitted elastic wave. We firstly investigated the wave attenuation characteristic of the system without a proof mass attached to the shunt resonators. We considered four sub-cases where the mechanical shunt resonators had dimensions of 6.25 × 20 × 0.1 mm^3^, 7.5 × 20 × 0.145 mm^3^, 8.75 × 20 × 0.197 mm^3^, and 10 × 20 × 0.257 mm^3^, respectively. The transmission diagrams of the system are presented in Figure 6. 

Figure 6a shows the transmission diagram of the system without a proof mass attached to the shunt resonators. Figure 6b presents the detailed transmission diagram in the frequency range of 2400–3200 Hz. The transmission diagram of an original beam, the one without mechanical shunt resonators integrated, is presented for a comparison. It can be observed that the conventional beam had no wave attenuation capability in the frequency range of interest. Integrating the mechanical shunt resonators introduced a strong elastic wave attenuation capability in the vicinity of the resonance frequency of 2.7 kHz. Besides, small-amplitude variations of transmission in the low-frequency range can be observed. This can be attributed to the imperfectly matched layers in the frequency range. 

**Figure 7:**
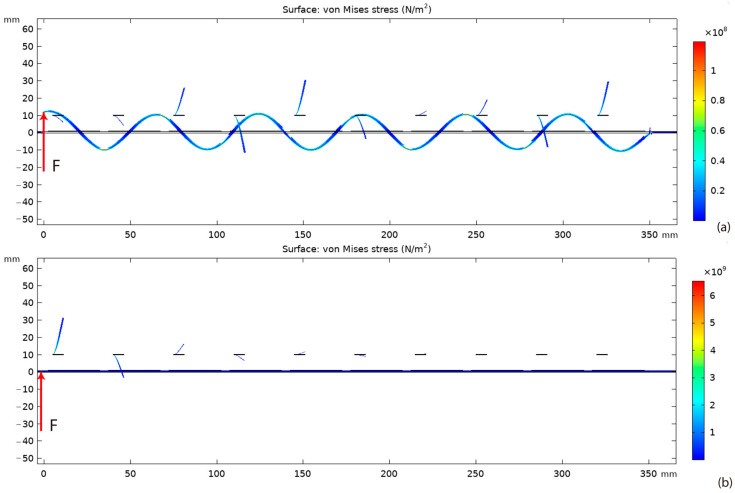
Displacement distributions of the system at different frequencies: (**a**) 2400 Hz; (**b**) 2730.6 Hz.

Moreover, the frequency responses of the system with shunt resonators with dimensions of 6.25 × 20 × 0.1 mm^3^, 7.5 × 20 × 0.145 mm^3^, 8.75 × 20 × 0.197 mm^3^, and 10 × 20 × 0.257 mm^3^ were analyzed. In addition, the proposed system had a frequency range of wave attenuation with the frequency widths of 11.759 Hz, 12.236 Hz, 12.544 Hz, and 12.68 Hz for the respective four sub-cases. The frequency range of wave attenuation was expanded with the increase of the length of the shunt resonator. This is because the increase of the length can effectively increase its capacitance and thereby enhance the influence of the dynamics of the shunt resonator towards the primary structure. It is worth noticing that the frequency of wave attenuation was lower than 3 kHz, the resonant frequency of a stand-alone shunt resonator. For example, the system with a shunt resonator having a dimension of 6.25 × 20 × 0.1 mm^3^ had the maximum wave attenuation at 2730.6 Hz. This phenomenon can be attributed to the fact that the coupled system, i.e., the coupled main beam and the shunt resonator, worked at the series resonant frequency. The displacement distributions of the system with a shunt resonator having a dimension of 6.25 × 20 × 0.1 mm^3^ outside and within the wave attenuation range are shown in Figure 7. 

Figure 7 shows the displacement distributions of the system at 2400 Hz and 2730.6 Hz. The excitations were applied at the left end of the main beam. It can be obtained that a minor wave attenuation effect can be observed at 2400 Hz, the frequency point outside the region of wave attenuation. In this case, the main beam had large-amplitude vibrations. In addition, all the mechanical shunt resonators were subjected to small-amplitude vibrations due to the coupling to the main beam. It can also be obtained that the main beam had a minor displacement at 2730.6 Hz although excitations with the same amplitude were applied. In other words, the elastic wave was attenuated significantly at this frequency point. Here, the first four remote shunt resonators had large-amplitude vibrations, while the following ones resonated with much smaller amplitudes. This is because the vibrations of the first four resonators provided a response force that prevented wave propagation in the main beam. It is noticeable that the results were obtained in a steady-state condition. In other words, the remaining resonators may have large-amplitude vibrations in suspending a transit elastic wave. In addition, it can be obtained that the length of the elastic wave was much larger than that of the unit-cell, i.e., the proposed system attenuated the wave in the sub-wavelength scale. 

It is worth mentioning that the wave attenuation capability of the proposed system stemmed from the impedance modification rather than the bandgap. In particular, the resonant dynamics of the shunt resonator affected the mechanical characteristics of the main beam significantly in the vicinity of the series resonance. Therefore, the mechanical impedance of the main beam varied dramatically, and hence, the elastic wave was reflected or temporally stored in the unit-cell. Hence, we had a strong wave attenuation capability of the system. On the other hand, the proposed system did not have a conventional bandgap. This is because the exitance of terms of $C_{p}^{\varepsilon }/C_{p2}$ and $C_{p}^{\varepsilon }/m$ in Equation (4) enables the dispersion equation with complex solutions in the entire frequency domain.

**Figure 8:**
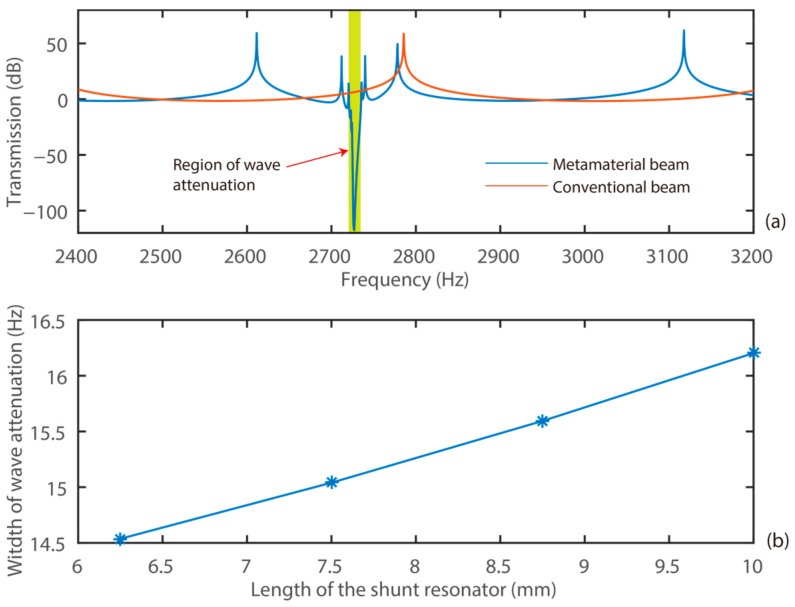
Characteristics of the system with a proof mass of 1 × 1 × 20 mm^3^: (**a**) transmission diagram; (**b**) frequency width of wave attenuation.

We further investigated the characteristics of the transmission of the proposed system with a proof mass attached to the mechanical shunt structure. Here, the copper proof mass had a dimension of 1 × 1 × 20 mm^3^. In addition, four sub-cases were considered, where the shunt resonators had dimensions of 6.25 × 20 × 0.191 mm^3^, 7.5 × 20 × 0.236 mm^3^, 8.75 × 20 × 0.288 mm^3^, and 10 × 20 × 0.346 mm^3^, respectively. Similarly, the resonant frequency of the shunt structure was kept as 3 kHz. The transmission diagrams of the systems are presented in Figure 8.

Figure 8 shows the transmission diagrams of the system with a proof mass attached to the shunt resonators. It can be observed that integrating the mechanical shunt resonators enabled strong elastic wave attenuation at 2730.3 kHz. Moreover, the frequency responses of the system with shunt resonators having dimensions of 6.25 × 20 × 0.191 mm^3^, 7.5 × 20 × 0.236 mm^3^, 8.75 × 20 × 0.288 mm^3^, 10 × 20 × 0.346 mm^3^, respectively, were obtained. Additionally, the proposed system had a frequency range of wave attenuation with the respective frequency widths of 14.537 Hz, 15.040 Hz, 15.59 Hz, and 16.202 Hz. That is, the introduction of the proof mass enlarged the frequency range of wave attenuation. For example, the system with a shunt resonator having a dimension of 10 × 20 × 0.346 mm^3^ and a 1 × 1 × 20 mm^3^ proof mass had a frequency range of wave attenuation enlarged by 27.7% than that of the one with a shunt resonator having a dimension of 10 × 20 × 0.257 mm^3^ and no proof mass. This trend followed that of the conventional mechanical metamaterial. 

**Figure 9:**
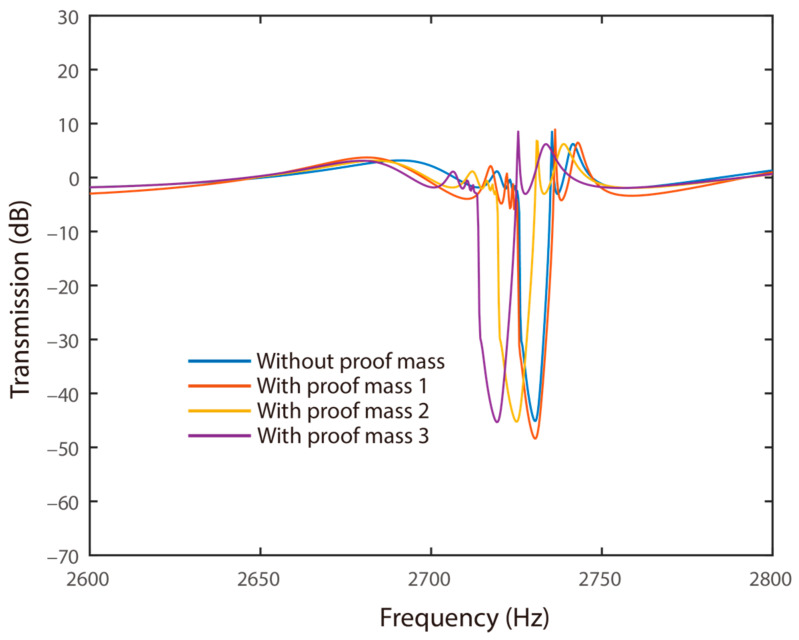
Transmission diagrams of the system with different proof masses.

To analyze the influences of the proof mass on the wave attenuation characteristics of the proposed system, four cases were evaluated. In the four cases, the shunt resonator had proof masses with dimensions of 0 mm^3^, 1 × 1 × 20 mm^3^, 1.5 × 1.5 × 20 mm^3^, and 2 × 2 × 20 mm^3^, respectively. Moreover, the lengths of the shunt resonators were kept as 6.25 mm, and their thicknesses were modified for creating the first resonance at 3 kHz. The corresponding transmission diagrams of the system are presented in Figure 9.

Figure 9 shows the transmission diagrams of the system with different proof masses attached to the shunt resonators. It can be obtained that the frequency range of wave attenuation was expanded by adding a large proof mass to the mechanical shunt resonators. In particular, the proposed system had a frequency range of wave attenuation with the frequency widths of 11.759 Hz, 14.537 Hz, 14.625 Hz, and 15.165 Hz for the four cases, respectively. That is, adding a proof mass with a dimension of a 2 × 2 × 20 mm^3^ can enlarge the frequency width of wave attenuation by 28.9%. It was illustrated that increasing the proof mass can effectively enlarge the frequency range of wave attenuation. This trend followed that of the effect of the mass ratio in conventional mass-in-mass mechanical metamaterials well, considering the electromechanical beams with periodic resonators. This phenomenon is similar to the flexural wave bandgap behaviors in metamaterial Timoshenko beams, analyzed by Miranda, where both locally resonant and Bragg-type bandgaps are enhanced by increasing the mass ratio [43]. Indeed, this phenomenon showed the linear characteristics of the proposed system. Introducing extra nonlinearity may further improve the performance of wave attenuation. In addition, it can be observed from Figure 9 that increasing the value of the proof mass would reduce the frequency range of wave attenuation. It can be attributed to the fact that adding mass would reduce the series resonant frequency of the coupled system.

**Figure 10:**
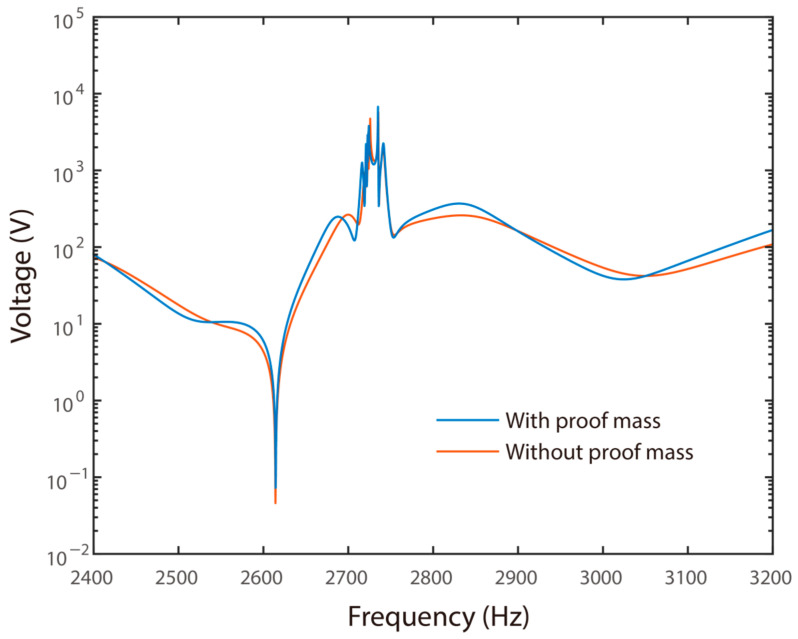
Frequency responses of the voltage without and with a proof mass.

The key advantage of the proposed system is the potential in overcoming the voltage limitation of conventional piezoelectric metamaterials while maintaining the compactness of the primary structure. Here, the voltage applied to the first piezoelectric transducer on the main beam was evaluated. Normally, the first piezoelectric transducer had the largest voltage in steady-state responses. Similarly, case studies were carried out, where the shunt resonators were with and without a proof mass. In the first case, a 1 × 1 × 20 mm^3^ proof mass was attached to the resonator. Besides, the dimensions of the shunt resonators followed those cases illustrated in Figure 6a and Figure 7a. The corresponding frequency responses of the voltage are presented in Figure 10. Here, the incident elastic wave had an amplitude of 0.1 mm.

Figure 10 shows the frequency responses of the voltage on the first piezoelectric transducer for the system without and with a proof mass attached to the shunt resonator. It can be obtained the frequency responses of the voltage had minor differences in the two cases. In addition, the frequency responses did not have a valley, although the transmission diagram had a deep valley indicating strong wave attenuations. It can also be obtained that high voltages exited in the frequency range of wave attenuation. Specifically, the peak voltages were 2736 V and 2725.5 V within the frequency range of wave attenuation in the two cases, respectively. 

**Figure 11:**
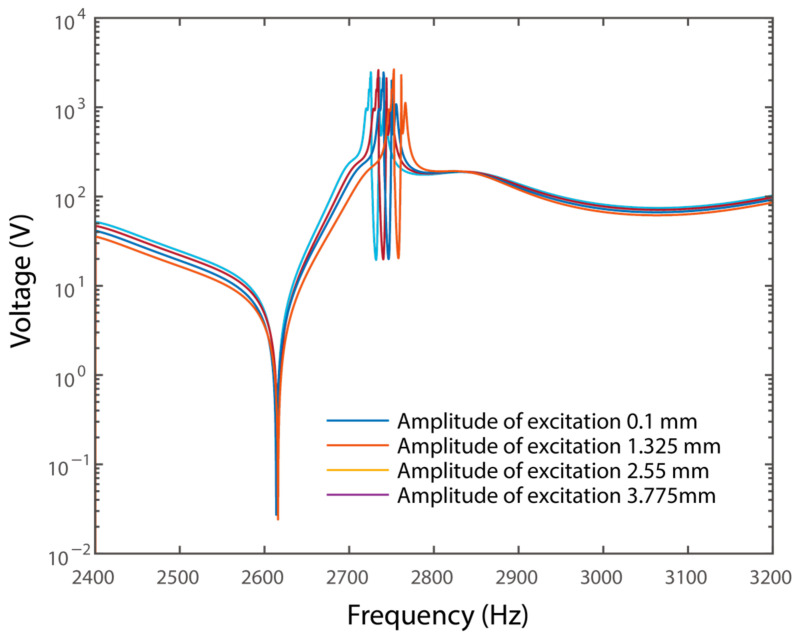
Frequency responses of the voltage under different amplitudes of excitation.

Normally, metamaterials were supposed to be applied in suspending large-amplitude vibrations. Hence, frequency responses under different amplitudes of excitations were evaluated for a further investigation of the characteristics of the system. In particular, bending waves with amplitudes of 0.1 mm, 1.325 mm, 2.55 mm, and 3.775 mm were considered. In addition, a 1 × 1 × 20 mm^3^ proof mass was attached to the resonator, and the dimension of the shunt resonator was chosen as 6.25 × 20 × 0.191 mm^3^. The frequency responses of the voltage are shown in Figure 11.

It can be obtained that the response of the voltage was increased dramatically with the increase of the amplitude of the excitation. For instance, the maximum voltage within the frequency range of wave attenuation was 2725.5 V, when the amplitude of excitation was 0.1 mm. Meanwhile, the voltage was increased to 2752.8 V, when the amplitude of excitation was 3.775 mm. It indicated that a high voltage was necessary to attenuate large-amplitude elastic waves. Such a high amplitude exceeded the voltage limitation of op-amps in conventional piezoelectric metamaterials significantly. For instance, the op-amp had a voltage limitation of ±15 V. Meanwhile, the high-voltage amplifier specially designed for the piezoelectric transducer, the OPA445, has a voltage limitation of ±45 V. However, such a high voltage would be within the voltage limitation with a proper selection of the piezoelectric transducer. A typical piezoelectric transducer holds a voltage of more than 2000 V/mm. More recently, a piezoelectric thin film made by TDK can hold a voltage up to 30,000 V/mm. Consider the fact that suspending large-amplitude excitations is sometimes necessary for real applications. Op-amp-based conventional piezoelectric metamaterials may break down easily in such a circumstance. The proposed system eliminates the usage of op-amps and creates a local resonance by using piezoelectric transducer-coupled mechanical shunt resonators. The proposed system shows advantages for the suspension of large-amplitude excitations.

## 5. Conclusions

In this research, we proposed a piezoelectric metamaterial with mechanical shunt resonators for elastic wave attenuations. Case studies using FEM simulations were designed, and the wave attenuation capability of the proposed system was analyzed. It was illustrated that significant wave attenuation can be obtained in the vicinity of the resonant frequency of the shunt resonators. Besides, increasing the proof mass of the mechanical shunt resonator can effectively enlarge the frequency range of wave attenuations. The proposed system has the potential in the attenuation of elastic waves with large amplitudes while maintaining the compactness of the primary structure. Moreover, we expect this approach can be extended to the design of two-dimensional structures with the same underlying physics. The proposed method can be used in many applications, including wave attenuation and vibration suspending in machines, vibration isolation platforms, and precision instruments. 

## Data Availability

Data Sharing is not applicable.
